# Noninvasive intracranial pressure monitoring for HIV-associated cryptococcal meningitis

**DOI:** 10.1590/1414-431X20176392

**Published:** 2017-08-07

**Authors:** V.R. Bollela, G. Frigieri, F.C. Vilar, D.L. Spavieri, F.J. Tallarico, G.M. Tallarico, R.A.P. Andrade, T.M. de Haes, O.M. Takayanagui, A.M. Catai, S. Mascarenhas

**Affiliations:** 1 Departamento de Clínica Médica, Faculdade de Medicina de Ribeirão Preto, Universidade de São Paulo, Ribeirão Preto, SP, Brasil; 2 Departamento de Neurociências e Ciências do Comportamento, Faculdade de Medicina de Ribeirão Preto, Universidade de São Paulo, Ribeirão Preto, SP, Brasil; 3 Braincare Health Technology, São Carlos, SP, Brasil; 4 Departamento de Fisioterapia, Universidade Federal de São Carlos, São Carlos, SP, Brasil

**Keywords:** Brain diseases, Intracranial hypertension, AIDS-related opportunistic infections, Cryptococcus neoformans, Monitoring

## Abstract

Mortality and adverse neurologic sequelae from HIV-associated cryptococcal meningitis (HIV-CM) remains high due to raised intracranial pressure (ICP) complications. Cerebrospinal fluid (CSF) high opening pressure occurs in more than 50% of HIV-CM patients. Repeated lumbar puncture with CSF drainage and external lumbar drainage might be required in the management of these patients. Usually, there is a high grade of uncertainty and the basis for clinical decisions regarding ICP hypertension tends to be from clinical findings (headache, nausea and vomiting), a low Glasgow coma scale score, and/or fundoscopic papilledema. Significant neurological decline can occur if elevated CSF pressures are inadequately managed. Various treatment strategies to address intracranial hypertension in this setting have been described, including: medical management, serial lumbar punctures, external lumbar and ventricular drain placement, and either ventricular or lumbar shunting. This study aims to evaluate the role of a non-invasive intracranial pressure (ICP-NI) monitoring in a critically ill HIV-CM patient.

## Introduction

A new ICP-NI method developed by Braincare™ Inc. (Brazil) has been used and evaluated to monitor patients with suspected intracranial hypertension, for example, in neurological hypertensive diseases, eclampsia and pre-eclampsia status, and infectious diseases leading to central nervous system hypertension. This system was tested in a patient with confirmed diagnosis of human immunodeficiency virus-associated cryptococcal meningitis (HIV-CM) and severe intracranial hypertension, before and after therapeutic lumbar puncture. This non-invasive measurement allows a real-time acquisition of ICP by the use of a sensor that acquires skull deformation data induced by the intracranial pressure waves (1). The waves and pulses of intracranial pressure reach the skull causing expansions and retractions in its volume. The Braincare sensor is able to detect these changes and convert them into numerical values that are interpreted in real time and shown as graphs for the interpretation of health professionals (2,3).

## Material and Methods

**Figure 1.**
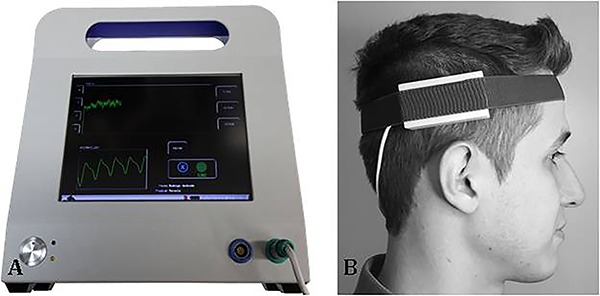
*A*, Braincare intracranial pressure (ICP) monitor 2000; *B*, Braincare ICP non-invasive sensor.

The sensor was positioned on the patient’s scalp without the need for trichotomy, surgical incision and trepanation (Figure 1). Noninvasiveness is of paramount importance in patients with HIV as it increases safety for the patients themselves and for all the healthcare professionals who are treating them.

Analysis of ICP morphology provides information about brain compliance and is correlated with ICP values (P). The increase in ICP and the consequent decrease in cerebral complacency lead to a change in ICP pulse morphology. Normal values show a morphology with P1>P2, but in patients with elevated ICP values the curve shows peaks with P2>P1 (4–6).

ICP morphology was acquired using the Braincare BCR-2000 ICP monitor and the analysis was performed with the Braincare Analytics System. After selecting the time-interval of interest, the software obtains the pulses of the average ICP with a confidence level of 95%, represented in figures as a grey region around the black line of the average ICP.

This project had institutional review board approval by the Ethics Committee of Hospital das Clínicas, Faculdade de Medicina de Ribeirão Preto, Universidade de São Paulo.

## Case Report

A 29-year-old male with a recent diagnosis of HIV and severe immunosuppressive status (CD4+T lymphocyte: 4 cells/mm^3^ and HIV viral load: 352,446 copies/mL) presented with headache and fever for 2 weeks, followed by altered mental status and seizures.

After initial investigation, cryptococcal meningitis was confirmed based on positive cerebrospinal fluid (CSF) India ink, positive agglutination test (CSF cryptococcal antigen titer >1:4096) and *Cryptococcus neoformans* CSF positive culture. The patient started liposomal amphotericin B medication and still presented with depressed consciousness level and agitation. Every other day, lumbar punctures were performed and showed opening pressures as high as 49 cmH_2_O (36 mmHg). The clinical status worsened slightly during the initial 10 days of amphotericin B.

On day 12 (D12), before and after a programmed lumbar puncture procedure, the patient underwent non-invasive intracranial pressure (ICP-NI) monitoring and all the usual CSF measurements and analysis. A second ICP-NI measurement was performed on day 34 (D34). The patient presented a slow and consistent recovery, reaching accumulated amphotericin B doses of 2050 g at D34, when the cryptococcal meningitis treatment was switched to fluconozale (600 mg qid), and the patient was discharged from the hospital.

## Results

The data related to the CSF analysis from day “zero” (D0) to day 34 (D34) are reported in Table 1.

**Table 1.** Cerebrospinal fluid (CSF) data of the patient from day zero (D0) until discharge from the hospital at day 34 (D34).

CSF analysis	Cell count (n/mL)	Lymph (%)	Protein (mg/dL)	Opening CFS pressure (cmH_2_O)	Closing CFS pressure (cmH_2_O)	India Ink	Culture
D0	61	99	143	40	nd	+	*C. neoformans*
D1	250	90	148	25	12	+	nd
D2	80	91	175	27	11	+	nd
D3	240	75	113	32	nd	+	nd
D4	nd	nd	nd	32	15	+	*C. neoformans*
D5	130	100	146	49	18	+	nd
**D12** *	**100**	**90**	**138**	**26**	**3.5**	**Negative**	**Negative**
**D34** #	**1.3**	**-**	**-**	**20**	**nd**	**Negative**	**Negative**

Lymph: lymphocyte percentage; nd: not done;

* Non-invasive intracranial pressure measurement;

# Accumulated liposomal amphotericin B (2050 g).

The patient presented with a typical CSF low cell count, with 99% of lymphocytes. The protein concentration was high and glucose level in CSF was low. The India ink and cryptococcal antigen test were positive and the final diagnosis was confirmed by *Cryptococcus neoformans* culture growth. During the first three weeks the patient’s CSF opening pressure was high, with an important increase in the first 12 days of treatment, followed by a slow and consistent decline in the next 2 weeks of intravenous antifungal treatment.

ICP-NI was monitored on day 12 (D12) and D34, always coupled with a programmed CSF lumbar puncture. Before and after D12 lumbar puncture, the patient underwent non-invasive intracranial pressure (ICP-NI) monitoring. The CSF opening pressure was 26 cmH_2_O (normal range 5 to 20 cm H_2_O); cell count was elevated at 100/µL, 90% lymphocytes; CSF glucose of 43 mg/dL (40–85 mg/dL) and elevated protein at 138 mg/dL (15–45 mg/dL). At the end of the procedure, CSF pressure was evaluated again and showed 3.5 cmH_2_O. During the ICP-NI monitoring, the patient was awake with spontaneous eye contact. He still maintained at least one fever peak/day (38°C) and breathed spontaneously with Cheyne-Stokes pattern. The neurological examination showed Glasgow Coma Scale = 13 (O: 4 V: 3 M: 6) and left side paresis.

**Figure 2.**
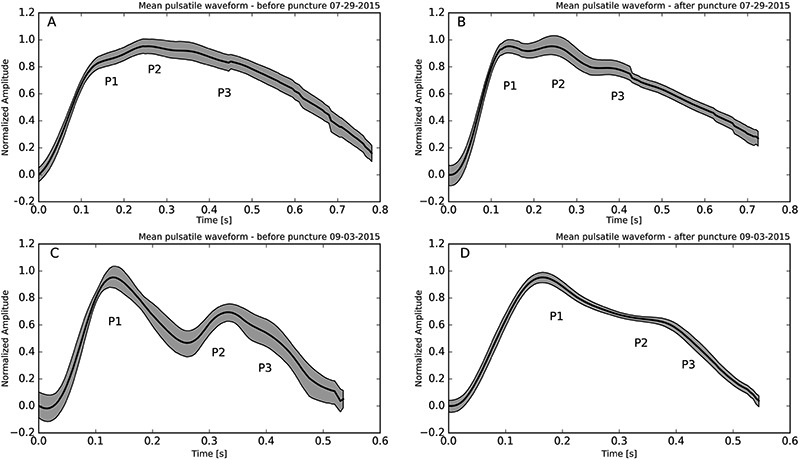
Normalized mean pulsatile waveform from non-invasive intracranial pressure (ICP-NI) measurement. *A*, Pulsatile waveform before lumbar puncture on D12 (P2>P1), showing the presence of neurological symptoms. *B*, Pulsatile waveform at D12 after lumbar puncture (P1>P2), showing improvement of neurological symptoms. Pulsatile waveform on D34 before (*C*) and after (*D*) lumbar puncture with P1>P2 in the final recovery phase, with a normal clinical and neurological physical exam. The black line represents the average and the gray shadows represent 95% confidence intervals (nonparametric bootstrap, α=0.05, N=1000).

The first ICP-NI measurement showed P2 peak larger than P1 (Figure 2A), which was reversed after the lumbar puncture procedure (Figure 2B), showing P1>P2 associated with patient clinical improvement as ICP diminished. In the second ICP-NI measurement (D34), the patient was totally recovered, just before hospital discharge, with a normal clinical and neurological physical exam. The ICP-NI measurement just before and after lumbar puncture showed an expected normal pulsatile waveform, with discrete improvement after lumbar puncture and the reduction of ICP.

## Discussion

In 2015, an estimated 36.9 million people globally were living with HIV and nearly 30 years after the advent of antiretroviral therapy, opportunistic infections of the central nervous system (CNS), such as cryptococcal meningitis, cerebral toxoplasmosis, and tuberculous meningitis, are a major cause of morbidity and mortality in HIV-positive individuals (7).

Cryptococcal meningitis is a fungal infection most commonly caused by *Cryptococcus neoformans* and more rarely by *Cryptococcus gattii*. Cryptococcus is an environmental yeast found in soil and bird droppings, typically acquired through inhalation of spores. Initial infection with *C. neoformans* can lead to a primary pulmonary infection, latent infection (asymptomatic cryptococcal antigenemia), or disseminated infection with a predilection for the CNS (8). Cryptococcal meningitis associated with HIV infection is estimated to cause more than 600,000 deaths each year, the vast majority in sub-Saharan Africa and in South and Southeast Asia (9).

Elevated CSF pressure is often seen in these patients. Significant neurological decline can occur if elevated CSF pressures are inadequately managed. Various treatment strategies to address intracranial hypertension in this setting have been described, including medical management, serial lumbar punctures, external lumbar and ventricular drain placement, and either ventricular or lumbar shunting. The literature remains unclear as to when each approach is indicated and how often patients require neurosurgical intervention, but there is no doubt about the importance of early diagnosis and adequate management of cryptococcal meningitis and severe intracranial hypertension (10).

ART in the setting of this opportunistic infection can lead to a paradoxical worsening caused by an immune reconstitution inflammatory syndrome (IRIS) leading to intracranial hypertension and all the possible complications (7). Among patients receiving antifungal therapy, mortality remains higher than 30% at 10 weeks, and survivors often have substantial disability, so there is a pressing need to improve outcomes. However, no new anti-cryptococcal agent is currently close to approval for clinical use and innovative strategies are needed (11).

In this report, we show a non-invasive intracranial pressure monitoring method as a new bedside strategy for real-time monitoring with high acquisition rate of intracranial pressure, which additionally provides unique valuable diagnostic medical evidence of intracranial hypertension and the immediate results of interventional procedures.

Currently, intracranial pressure monitoring is done using invasive sensors inserted inside the skull to detect the pressure and its variation over time (12). The invasiveness and high cost of this technique make its use not accessible for most of the patients. Studies in the last decades have shown that the analysis of the morphology and the tendency of the ICP over time bring important information about the clinical picture of the patient and mainly about the state of the cerebral complacency of the individual (13,14). Monitoring of intracranial pressure by noninvasive means is being developed by various groups around the world using technologies such as ultrasound (15), transcranial Doppler (16) and imaging (17–19). These strategies are experimental and they are not yet standardized and available in the market.

The ICP-NI sensor was able to monitor the pulses of intracranial pressure, showing morphological changes that were consistent with the patient’s clinical status. The initial findings motivated the beginning of a study on the application of the new sensor in patients with infectious diseases of the central nervous system. The method used here presented practicality, safety and reproducibility in its measurements, essential characteristics for the proposed use. The low cost of monitoring is another important differential of this methodology, making its use in hospitals of the public health system possible.
